# Influence of Socioeconomic Status Trajectories on Innate Immune Responsiveness in Children

**DOI:** 10.1371/journal.pone.0038669

**Published:** 2012-06-07

**Authors:** Meghan B. Azad, Yuri Lissitsyn, Gregory E. Miller, Allan B. Becker, Kent T. HayGlass, Anita L. Kozyrskyj

**Affiliations:** 1 Women and Children's Health Research Institute, Department of Pediatrics, University of Alberta, Edmonton, Alberta, Canada; 2 Department of Immunology, University of Manitoba, Winnipeg, Manitoba, Canada; 3 Department of Psychology, University of British Columbia, Vancouver, British Colombia, Canada; 4 Department of Pediatrics and Child Health, University of Manitoba, Winnipeg, Manitoba, Canada; South Texas Veterans Health Care System and University Health Science Center San Antonio, United States of America

## Abstract

**Objectives:**

Lower socioeconomic status (SES) is consistently associated with poor health, yet little is known about the biological mechanisms underlying this inequality. In children, we examined the impact of early-life SES trajectories on the intensity of global innate immune activation, recognizing that excessive activation can be a precursor to inflammation and chronic disease.

**Methods:**

Stimulated interleukin-6 production, a measure of immune responsiveness, was analyzed *ex vivo* for 267 Canadian schoolchildren from a 1995 birth cohort in Manitoba, Canada. Childhood SES trajectories were determined from parent-reported housing data using a longitudinal latent-class modeling technique. Multivariate regression was conducted with adjustment for potential confounders.

**Results:**

SES was inversely associated with innate immune responsiveness (p = 0.003), with persistently low-SES children exhibiting responses more than twice as intense as their high-SES counterparts. Despite initially lower SES, responses from children experiencing increasing SES trajectories throughout childhood were indistinguishable from high-SES children. Low-SES effects were strongest among overweight children (p<0.01). Independent of SES trajectories, immune responsiveness was increased in First Nations children (p<0.05) and urban children with atopic asthma (p<0.01).

**Conclusions:**

These results implicate differential immune activation in the association between SES and clinical outcomes, and broadly imply that SES interventions during childhood could limit or reverse the damaging biological effects of exposure to poverty during the preschool years.

## Introduction

Social environments in early life are known to affect the course of human development. Socioeconomic status (SES) differences originating in childhood can influence susceptibility to cardiovascular disease, diabetes, asthma, and obesity in later life [1], [2]. Lower SES has consistently been associated with greater risk of disease, more rapid progression, and decreased survival [3]; however, since most studies focus on adult outcomes, it is unclear *when* SES-related health disparities emerge during the life course. In addition, despite longstanding and worldwide recognition of social gradients in health, the biological mechanisms underlying these inequalities remain poorly understood.

SES is a complex and dynamic measure reflecting gradients of exposure to various environmental and lifestyle factors, such as neighbourhood crime, air pollution, family stress, and access to healthcare and proper nutrition. As a multi-dimensional construct, SES can be challenging to quantify for research purposes. Commonly used SES indicators vary from “wealth” measures such as income and savings, to “prestige” measures such as occupation or education [1]. SES can also be conceptualized at the individual, family or neighbourhood level, with each sphere having somewhat distinct relationships with health. While the great majority of studies employ static measures of SES, reflecting a single point in time, there is growing interest in the cumulative effect of SES beginning in early life [1]. Such dynamic, “trajectory” measures provide invaluable information [4] but are uncommon because data are often lacking to implement them.

One mechanism by which SES may ultimately exert its effects on health is via differential activation of the immune system, thereby influencing the development of inflammatory conditions. Inflammation is a key pathway in many diseases including rheumatoid arthritis, cardiovascular and neurodegenerative diseases, asthma, diabetes, obesity and cancer [5], [6]. Extensive cross sectional studies link low SES to increased systemic immune activation, inflammation, poor health outcomes, and reduced activity of the anti-inflammatory hormone cortisol [7]–[9]. While the causal role of immune activation remains controversial [10]–[12], SES-related differences are documented for pro-inflammatory gene expression [13], [14] and diverse circulating inflammatory biomarkers such as C-reactive protein and interleukin-6 (IL-6) [15]–[19]. IL-6 levels are widely used as a surrogate marker of chronic inflammation; however, this molecule can function as a pro-inflammatory or anti-inflammatory cytokine depending on the physiological context. IL-6 also exhibits highly pleiotropic functions in immune regulation, inflammation and oncogenesis among others. Thus, tagging IL-6 as a biomarker of inflammation risks over-simplification of a molecule exhibiting a broad range of activities depending on the stimulus and outcome examined [20]–[22]. Here, we assess IL-6 as a global indicator of the intensity of innate immune capacity or activation, rather than attributing a single specific quality to the response.

It is important to note that many circulating biomarkers, including plasma IL-6 levels, fluctuate substantially according to meal consumption, time of day, exercise and the menstrual cycle [23]. By contrast, we find that IL-6 responses elicited directly *ex vivo* by environmental stimuli, while considerably more laborious than plasma analyses, can be standardized to yield reliable measures of innate immune capacity in any given individual [24]. Moreover, IL-6 originates *in vivo* from multiple sources beyond immune cells, including adipose tissue, muscle and the central nervous system among others [25]. Consequently, circulating IL-6 does not necessarily reflect immune activation, but there is no such ambiguity with an *ex vivo* stimulated measure.

Thus, previous studies seeking to identify associations between SES, immune biomarkers and clinical outcomes have been challenged to account for changes in SES, have been primarily conducted in adults, and have mainly analyzed circulating biomarkers susceptible to considerable experimental challenges. In this study, undertaken with an appreciation for SES as a multi-dimensional construct, we aimed to determine if associations were detectable between proxy dynamic measures of SES throughout childhood, and global innate immune responsiveness at school age.

## Methods

### Ethics Statement

The study was approved by the University of Manitoba Health Research Ethics Board and Health Information Privacy Committee. Parents also provided written informed consent.

### Study population

The study population comprised 267 children from the nested case-control arm of the Study of Asthma, Genes and Environment (SAGE) birth cohort in Manitoba, Canada. Full details of the study design are reported elsewhere [26]. Based on estimates from our previous research [24] (mean log-normalized IL-6 response, in pg/mL, upon 0.05 ng/mL LPS stimulation = 7.0; standard deviation = 0.5) we calculated that the required sample size to detect a 50% change in absolute IL-6 production, with 5% error and 80% power, is 24 subjects per SES trajectory group.

Children were recruited at mean age 9 (range 8–10) after parent response to a mailed survey on child health; they were examined by a pediatric allergist to confirm asthma status, and atopy was determined by skin-prick testing for common allergens [26]. Overweight was defined as body mass index ≥85th percentile according to age and sex-specific growth charts [27]. Maternal asthma was defined as report of: asthma or use of asthma medications in the last 12 months, or physician-diagnosed asthma with onset after age 5. Maternal allergy was defined as report of hay fever or food allergy. Data on family and environmental factors were obtained from the same questionnaire, including: family size (number of people living in the family home, including study child, at age 9), number and age of siblings, residence location (urban or rural, based on home postal code), household income (reported in several categories), relocation since birth (ever or never), First Nations ethnicity (FN; also known as Canadian aboriginal), gestational age, birth weight, breastfeeding (defined as exclusive breastfeeding for at least 3 months), and maternal smoking (during pregnancy and/or the first year after birth). Perinatal maternal distress (present or absent) was defined based on mother's response to a question on whether she felt down, depressed or hopeless during the year after delivery, as previously described [28]. Small-for-gestational-age (SGA) was defined as <10^th^ percentile according to sex-specific percentiles for birth weight and gestational age in Canadian children [29]. Child use of anti-inflammatory medications (such as beclomethasone, Pulmicort, or Flovent) in the preceding 12 months was also reported by parents.

Due to limited resources for processing fresh blood, not all case-control children could be assessed for LPS-stimulated IL-6 production. Nevertheless, a large subgroup was selected (N = 409), including 164 healthy children without asthma or allergies. Analyses were conducted on the 267 children with SES trajectory data (see below). Children with known versus unknown SES trajectories did not differ on current household income, IL-6 production, family size, gender, atopy or asthma (Table 1).

**Table 1:** Characteristics of children with known versus unknown SES trajectories.

	SES Trajectory		
	unknown	known	
	n = 139	n = 267	p^3^
**Gender** (% male)	54.0	55.4	0.77
**First Nations Ethnicity** (% yes)	35.3	16.9	0.001
**Residence** (% rural)	58.3	43.8	0.01
**Child Overweight** (% yes)	41.0	25.8	0.001
**Child Atopy/Asthma** (%)			
Atopic Asthma	20.1	21.7	0.60
Atopy, no Asthma	25.2	24.0	
Non-atopic Asthma	17.3	12.7	
Healthy	37.4	41.6	
**Anti-Inflammatory Medications** (%yes)	26.6	31.5	0.31
**Mother Allergy/Asthma** (% yes)	48.9	63.3	0.01
**Annual Family Income** (%)			
<$50,000	32.4	24.5	0.27
<$80,000	34.3	35.7	
≥$80,000	33.3	39.8	
**Older Maternal Siblings** (% yes)	54.0	58.0	0.45
**Birth Weight** (% Preterm or SGA)	21.1	18.3	0.52
**Exclusive Breastfeeding** 1 (% yes)	40.0	54.7	0.01
**Mother Smoked** 2 (% yes)	30.2	19.1	0.01
**Maternal Distress** (% yes)	19.6	22.1	0.60
**Family Size at age 9** (mean)	4.7	4.6	0.29
**IL-6** (geometric mean, pg/mL)	1138	1097	0.74

IL-6, interleukin-6; SES, socioeconomic status; SGA, small for gestational age.

IL-6 production induced by LPS treatment of PBMC.

1 Exclusive breastfeeding ≥3 months;

2 Mother smoked during pregnancy and/or first year after birth; ^3^Comparisons by chi-squared test (categorical variables) or t-test (continuous variables).

### SES trajectories

Using the method recently reported by Marin et al. [30], childhood SES trajectories were derived from questionnaire data where parents reported the number of bedrooms in the family home for each year of the child's life. Number of bedrooms was used as a proxy for SES because housing is more accurately recalled compared to other measures such as income [31], and is a more dynamic measure compared to indicators such as parent education. Thus, since longitudinal income data were not collected prospectively and cannot be accurately recalled retrospectively, number of bedrooms was selected as a reliable and dynamic measure which could be used to study SES trajectories throughout childhood. Most families in our study (55%) had moved since the child's birth, allowing us to capture changes in housing over time. Marin et al. [30] established that “number of bedrooms” is a better indicator of family wealth than “number of bedrooms per person”, a measure of crowding best used to distinguish individuals at the low end of the SES spectrum. Nevertheless, to account for family size as a potential confounder, we adjusted all statistical models for family size (number of people living in the family home) at age 9.

A semiparametric, group-based modeling strategy (the SAS “TRAJ” procedure) was used to separate individual children into trajectory groups [32], based on changes to the number of bedrooms in the family home throughout childhood. Bayes' theorem was coupled with TRAJ to assign children to the SES/bedroom trajectory group to which they had the highest probability of belonging. The desired number of trajectories (beginning with a single trajectory and increasing one at a time) was specified before running each model. The Bayesian Information Criterion (BIC) was used to select the final number of trajectories, with the goal of identifying the fewest number of trajectories that best fit the data [32], [33].

### Cell culture and IL-6 assay

Whole blood was drawn during a daytime clinic visit at mean age 9. The time was recorded, and processing was initiated within 3 hours. All subjects reported being free of symptomatic respiratory infections in the preceding two months. Peripheral blood mononuclear cells (PBMC) were isolated from whole blood by standard techniques and used immediately for culture in triplicate. Preliminary studies were performed to establish optimal culture conditions, cell density and time of harvest (not shown). We previously demonstrated the importance of utilizing environmental immune stimuli at physiologic levels [24]; therefore, PBMC were stimulated with 0.05 ng/ml bacterial endotoxin (lipopolysaccharide, LPS) (Sigma). Supernatants were harvested after 24 hours and IL-6 production was quantified by ELISA (Biolegend). Each sample was evaluated in at least 2 assays, with the IL-6 concentration calculated from a minimum of 3 points falling on the linear portion of standardized titration curves. Standard errors typically ranged from 3–10% per sample. The time of blood draw did not influence IL-6 production (r = −0.02, p = 0.82).

### Statistical analysis

Statistical analyses were conducted using SAS 9.2 statistical software package and GraphPad Prism 5 software. IL-6 values were normalized by log-transformation and compared by t-test, univariate linear regression, or ANOVA with Bonferroni's post-hoc test for multiple comparisons. Distribution among SES trajectory groups was compared by chi-squared test.

Multiple linear regression was conducted to adjust associations between SES trajectories and IL-6 for the following covariates, based on established associations with inflammation or IL-6 production: residence location [34], [35], child atopy/asthma phenotype [36]–[38], maternal history of allergy/asthma [39], [40], overweight [41], gender [42], and FN ethnicity [43]. Early life factors known to influence inflammation or IL6 were tested in secondary analyses; these included: maternal distress [44], maternal smoking [45], mother's parity [46], exclusive breast feeding >3 months [47], birth weight [48], and relocation since birth [49]. All models were adjusted for family size at age 9. Interaction terms were tested using a relaxed type 1 error rate of 10%, a common approach in epidemiological studies used to increase power for detecting effect modification [50]. Results are reported as relative differences (response ratios) and associated p-values.

## Results

### Study population

The 267 study participants (55% male; mean age 9) were distributed between urban (56%) and rural (44%) residence locations, and included 45 (16.9%) First Nations (FN) children (Table 2). Similar to the Manitoba population, the remaining children were predominantly Caucasian (76.0%). By design, the study over-sampled children with asthma; there were 58 children with atopic asthma, 34 with non-atopic asthma, 64 with atopy only, and 111 healthy children. Twenty-six percent of children were overweight.

**Table 2:** Population characteristics and distribution of potential confounding factors in relation to innate immune responsiveness (stimulated IL-6 response) at age 9 and childhood SES trajectories.

			IL-6 Response (pg/ml)		Childhood SES Trajectory Distribution (%)					
		N (%)	Geo. Mean	p^4^	Low	Mid	Mid-High	High	Incre-asing	p^6^
**Overall**		267	1097		12.4	44.6	26.6	6.7	9.7	
**Gender**	Female	119 (44.6)	1087	0.92	7.6	47.9	27.7	5.9	10.9	0.27
	Male	148 (55.4)	1103		16.2	41.9	25.7	7.4	8.8	
**First Nations Ethnicity**	No	222 (83.2)	1054	0.14	10.4	46.9	26.6	6.3	9.9	0.18
	Yes	45 (16.9)	1331		22.2	33.3	26.7	8.9	8.9	
**Residence**	Urban	150 (56.2)	1258	0.03*	14.0	46.0	26.0	6.7	7.3	0.56
	Rural	117 (43.8)	920		10.3	42.7	27.4	6.8	12.8	
**Relocation Since Birth**	No	120 (44.9)	1277	0.05*	9.2	49.2	29.2	9.2	3.3	0.006*
	Yes	147 (55.1)	968		15	40.8	24.5	4.8	15.0	
**Child Overweight**	No	198 (74.2)	1152	0.23	9.1	45.0	28.3	8.1	9.6	0.05*
	Yes	69 (25.8)	951		21.7	43.5	21.7	2.9	10.1	
**Child Atopy/Asthma**	Atopic Asthma	58 (21.7)	1412	0.08	12.1	41.4	36.2	0.0	10.3	0.28
	Atopy Only	64 (24)	1065	0.82	17.2	46.9	20.3	7.8	7.8	
	Asthma Only	34 (12.7)	941	0.71	5.9	44.1	20.6	11.8	17.7	
	Healthy^5^	111 (41.6)	1024		11.7	45.1	27.0	8.1	8.1	
**Anti-Inflammatory Meds** 1	No	183 (68.5)	1047	0.30	13.1	42.6	8.2	26.8	9.3	0.10
	Yes	84 (31.5)	1211		10.7	46.8	13.1	26.2	1.2	
**Mother Allergy/Asthma**	No	97 (36.3)	1225	0.24	13.4	39.2	25.8	9.3	12.4	0.43
	Yes	167 (62.6)	1032		10.8	47.9	27.5	5.4	8.4	
**Annual Family Income**	<$50,000	61 (22.9)	1182	0.35	19.7	49.2	14.8	6.6	9.8	0.16
	<$80,000	89 (33.3)	1099	0.54	13.5	42.7	31.5	3.4	9.0	
	≥$80,000^5^	99 (37.1)	992		7.1	45.5	28.3	9.1	10.1	
**SES Trajectory**	Low	33 (12.4)	1994	0.02*						
	Mid^5^	119 (44.6)	1174							
	Increasing	26 (9.7)	728	0.05*						
	Mid-High	71 (26.6)	935	0.18						
	High	18 (6.7)	787	0.16						
**Older Maternal Siblings**	No	111 (41.6)	1089	0.94	17.1	53.2	18	3.6	8.1	0.003*
	Yes	153 (57.3)	1100		9.2	37.3	33.3	9.2	11.1	
**Birth Weight**	Normal	214 (80.2)	1119	0.68	11.7	47.2	24.3	7.9	8.9	0.21
	Preterm or SGA	48 (18)	1038		14.6	35.4	35.4	2.1	12.5	
**Exclusive Breastfeeding** 2	No	120 (44.9)	1112	0.94	15	44.2	26.7	5.8	8.3	0.75
	Yes	145 (54.3)	1101		10.3	44.8	26.2	7.6	11	
**Mother Smoked** 3	No	209 (78.3)	1097	1.00	10.5	46.4	29.2	5.7	8.1	0.05*
	Yes	58 (21.7)	1096		19	37.9	17.2	10.3	15.5	
**Maternal Distress**	No	201 (75.3)	1059	0.41	9	44.8	29.9	6	10.5	0.08
	Yes	57 (21.4)	1222		19.3	47.4	15.8	8.8	8.8	

IL-6, interleukin-6; Geo. Mean, geometric mean; SES, socioeconomic status; SGA, small for gestational age.

IL-6 production induced by LPS treatment of PBMC.

1 Anti-inflammatory medications used in the previous 12 months;

2 Exclusive breastfeeding ≥3 months;

3 Mother smoked during pregnancy and/or first year after birth;

4 Log-transofrmed IL-6 values compared by t-test (2-groups) or univariate regression (3+ groups);

5 reference group for multiple comparisons;

6 SES distributions compared by chi-squared test;

* p<.05.

### Identification of SES trajectory groups

**Figure 1:**
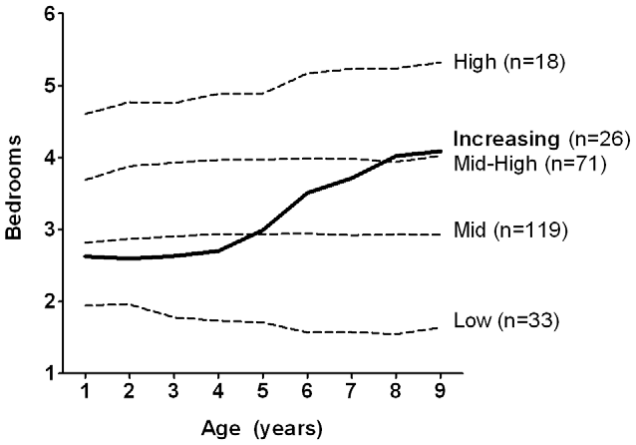
Childhood SES trajectories. SES trajectories were derived from parent-reported housing data (number of bedrooms in the family home, for each year of the child's life). Individual children were assigned to trajectory groups using a semiparametric, group-based statistical modeling strategy; the number of children in each group is shown. Plotted lines represent the average trajectory for each group. SES, socioeconomic status.

SES trajectories were derived from parent-reported housing data. As described in “Methods”, individual children were assigned to trajectory groups using a group-based statistical modeling strategy. The Bayesian Information Criterion (BIC) was −6096 for 3 groups, −5626 for 4 groups, and −5452 for 5 groups, demonstrating increasingly better fit with the addition of each trajectory group. To prevent small group size, we did not exceed a 5-group model. As shown in Figure 1, four of the five identified trajectories indicated stable SES throughout childhood (designated low, mid, mid-high, or high). The fifth “increasing SES” trajectory, where SES until age 4 was below the mid-SES group but improved to exceed the mid-high-SES group by age 9, represented a significant increase in SES from birth (p<0.001). The persistent mid-SES group was used as the reference group as it comprised the largest proportion of children (N = 119, 44.6%), thus representing the “average” childhood SES trajectory in this population-based cohort. The remaining children were classified as follows: 33 low-SES, 26 increasing-SES, 71 mid-high-SES, and 18 high-SES.

### Impact of SES trajectories on immune responsiveness

**Figure 2:**
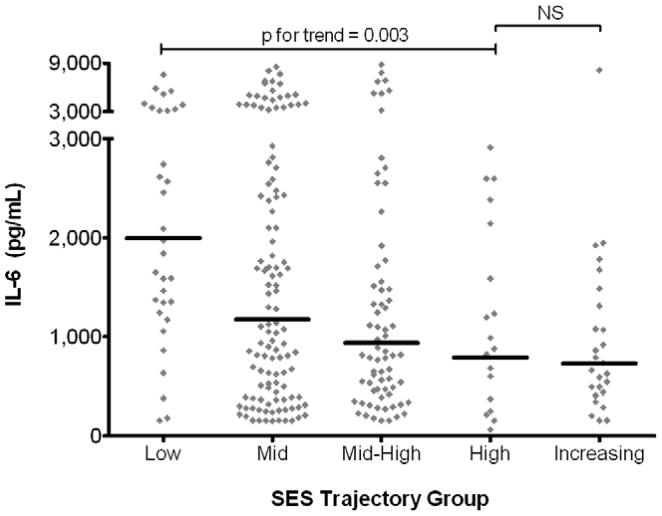
IL-6 responses by SES trajectory group. IL-6 responses were measured in LPS-stimulated PBMC by ELISA; children were assigned to SES trajectory groups as depicted in Figure 1. Data points represent individual children; bars represent geometric means. Log-normalized values compared by ANOVA with post-testing for linear trend and multiple comparisons. ELISA, enzyme-linked immunosorbent assay; LPS, lipopolysachharide; PBMC, peripheral blood mononuclear cells; SES, socioeconomic status.

IL-6 responses were quantified in freshly isolated immune cells following stimulation with LPS, a widely-encountered environmental stimulus of innate immune activation. Responses ranged from 10 to 10,000 pg/ml (Figure 2) with individual variation <10%, indicating the stability of this measure. A clear trend of elevated immune activation was found with decreasing SES (p for trend = 0.003). Persistent low-SES was associated with the highest IL-6 responses (geometric mean, 1994 pg/ml), while persistent high-SES children exhibited responses less than half this intensity (787 pg/ml). These data clearly indicate the propensity of high-SES children to exhibit more controlled immune activation upon stimulation with a ubiquitous environmental stimulus.

Importantly, we found that children in the increasing-SES trajectory group exhibited the lowest IL-6 responses (723 pg/ml), at a value not statistically different from that of the highest SES children. Thus, improving SES appears confer the same advantages as being a member of the highest SES trajectory group from birth. Current household income was not significantly associated with intensity of IL-6 production (Table 2), highlighting the advantage of using the more informative trajectory measure to capture changes in SES over time.

In unadjusted analyses (Table 2), persistent low-SES was associated with increased likelihood of FN ethnicity, overweight, low current household income, being firstborn, relocating during childhood, and having a mother who smoked or experienced perinatal distress. IL-6 production was significantly increased in urban children and tended to be higher in FN children and children with atopic asthma. Early life factors (such as birth weight, breastfeeding, and maternal smoking, distress and parity) and anti-inflammatory medication use were not associated with IL-6 production at age 9. Collectively, these findings demonstrate that a number of potentially inter-related demographic factors, including SES trajectories, are associated with the intensity of IL-6 production in school-aged children.

To determine the unique effects of SES trajectories on immune capacity, we conducted multivariate regression analyses (Table 3). The effect of SES trajectories on IL-6 production was found to be independent of FN ethnicity, residence location, atopic asthma, and family size. However, the SES trajectory/IL-6 association was influenced by overweight status (p for interaction = 0.08), such that the highest IL-6 responses were observed among children who were overweight and low-SES, while the lowest responses were observed in normal weight, high-SES or increasing-SES children. More specifically: among overweight children, the median level of IL-6 production was 3.1-fold higher in those who were low-SES, compared to their mid-SES counterparts (p = 0.001), whereas low-SES effects were non-significant in normal weight children (1.1-fold difference, p = 0.73). By contrast, the high- and increasing-SES trajectories were associated with particularly low IL-6 production for normal weight children only (nearly 50% lower than mid-SES children, p<0.05). As indicated above, these comparisons were adjusted for FN ethnicity, residence location, atopic asthma, and family size (Table 3, Model A).

**Table 3:** Innate immune responsiveness at age 9 according to childhood SES trajectories and other factors: IL-6 response ratios (95% CI).

		Model A		Model B	
Child Overweight		Normal	Overweight	Normal	Overweight
**SES Trajectory Group**	low	1.10 (0.63–1.92)	3.10 (1.57–6.14)**	1.10 (0.61–1.99)	3.11 (1.55–6.24)**
	mid	1.00 (ref)	1.00 (ref)	1.00 (ref)	1.00 (ref)
	mid-high	0.69 (0.48–1.00)*	0.94 (0.48–1.86)	0.63 (0.43–0.93)*	1.05 (0.51–2.13)
	high	0.53 (0.29–0.96)*	1.93 (0.39–9.49)	0.46 (0.25–0.86)*	1.72 (0.33–8.96)
	increasing	0.52 (0.30–0.90)*	1.21 (0.49–2.98)	0.46 (0.25–0.83)*	1.35 (0.54–3.37)
**First Nations Ethnicity**	yes	1.51 (1.02–2.23)*	1.51 (1.02–2.23)*	1.62 (1.12–2.61)*	1.62 (1.12–2.61)*
	no	1.00 (ref)	1.00 (ref)	1.00 (ref)	1.00 (ref)
**Residence/Atopic Asthma**	urban/yes	1.80 (1.20–2.72)**	1.80 (1.20–2.72)**	1.71 (1.12–2.61)*	1.71 (1.12–2.61)*
	urban/no	1.14 (0.83–1.57)	1.14 (0.83–1.57)	1.11 (0.79–1.55)	1.11 (0.79–1.55)
	rural/yes	0.77 (0.42–1.39)	0.77 (0.42–1.39)	0.69 (0.37–1.29)	0.69 (0.37–1.29)
	rural/no	1.00 (ref)	1.00 (ref)	1.00 (ref)	1.00 (ref)
**Also Adjusted For:**		family size at age 9	family size at age 9, maternal allergy/asthma, relocation since birth, and early life factors: breastfeeding, birth weight, parity		

SES, socioeconomic status; IL-6, interleukin 6.

Response ratios represent relative differences (ratios) of IL-6 responses compared to the appropriate referent (ref) category. For example, the value of 1.51 for First Nations children in Model A indicates that IL-6 responses were 51% higher compared to non-First Nations children. A value of 1.00 indicates no difference in IL-6 responses; values less than 1.00 indicate lower IL-6 responses (e.g., 0.90 would indicate a value of 10% lower for the group in question).

* p<.05;

** p<.01.

We performed further model adjustment for early life factors previously linked to innate immune activation: maternal parity, breastfeeding, birth weight, and relocation during childhood [46]–[49] (Table 3, Model B). Here, none were associated with IL-6 responses at age 9, nor did they significantly alter any of the associations described above Adjustment for gender, anti-inflammatory medication use, maternal distress, and maternal smoking also did not affect the results (data not shown). Notably, maternal history of allergy/asthma intensified the observed SES trajectory effects (p value for interaction = 0.03, results not shown), such that especially high IL-6 responses were elicited by low-SES, overweight children with maternal allergy/asthma, while especially low responses were elicited by increasing-SES, normal weight children without maternal allergy/asthma.

### Additional predictors of immune responsiveness

Multivariate analysis further identified an association between IL-6 production, residence location and atopic asthma (p for interaction = 0.03) – a notable finding given the extensive literature on urban-rural differences in inflammatory disease [34], [35]. In urban centers, atopic asthma was associated with a 54% increase in the intensity of IL-6 production (p = 0.03), whereas atopic asthma was not associated with IL-6 production in rural children (p = 0.24). These associations were independent of SES trajectories, overweight, and all other factors tested (Table 3, Model B). Finally, given the disproportionately low SES of Canadian FN in this region, and the increased prevalence of inflammatory disorders in this population [51], [52], we examined whether there was an association between FN ethnicity and immune capacity, independent of SES trajectories (Table 3). Indeed, FN children had 62% higher median IL-6 responses compared to non-FN children, regardless of SES trajectories and all other factors tested (p = 0.02).

## Discussion

In a cohort of 267 Canadian children born in 1995, we found evidence of elevated innate immune responsiveness in schoolchildren experiencing persistent low-SES since birth, consistent with the hypothesis that early social inequalities impact physiologic programming [1]. Our results are novel in several ways. First, we performed our analyses in children, whereas previous research has focused on clinical outcomes in adulthood. Second, we assessed stimulated IL-6 responses, whereas most previous studies have reported on circulating IL-6. Third, we found that the detrimental effect of low SES on immune responsiveness is exacerbated by overweight. Finally, and most importantly, by examining dynamic SES trajectories we identified a clear difference in immune responsiveness among those children who experienced increasing SES during early life.

This is the first study to investigate the relationship between dynamic SES trajectories and innate immune responsiveness in children. Overall, we observed that children living continuously in low SES environments had 70% higher IL-6 responses compared to children in mid-SES households. This elevation may be clinically significant, since equivalent increases in circulating IL-6 (in healthy adults) have been shown to predict myocardial infarction [53]. Previous studies have documented elevated IL-6 in adults who experienced low early-life SES [13], [54], but to date only one such study has been conducted in children [41]. In that report, Howe et al. reported socioeconomic inequalities in cardiovascular risk factors (including IL-6) in 10-year-olds, but they assessed IL-6 in serum and used “snapshot” measures of SES, whereas we measured immune responsiveness and characterized SES trajectories throughout childhood.

Overweight was found to intensify SES differences in immune capacity. Overweight, persistently low-SES children had 3-fold higher IL-6 responses compared to their mid-SES counterparts. Together with recent reports of overweight/SES interactions in asthma [55] and cardiovascular health [41], this suggests that overweight may play a preeminent role in moderating or amplifying SES effects on immune responsiveness and ultimate development of inflammatory disease. While additional research is required to determine the biological pathways behind these observations, our results point towards shared characteristics of low-SES and overweight children as mediators of immune responsiveness. For example, unhealthy diet and inactivity are independently associated with both low SES and overweight [56], and both can contribute to chronic inflammation [57], [58]. We therefore speculate that among low-SES children, those who are overweight have especially poor nutrition and low physical activity, resulting in especially elevated immune response profiles. The potential for overweight and low-SES to act synergistically in elevating immune responsiveness has considerable public health implications, since overweight and obesity disproportionately affect low SES populations [56].

Interestingly, the lowest intensity of inflammatory IL-6 responses occurred in children of the increasing-SES trajectory group, who produced levels that were indistinguishable from children at the highest SES level. They were also clearly distinct from the mid-SES group, despite being born into a similar socioeconomic environment. Consistent with evidence for the detrimental influence of low SES during early life [30], and for a protective role of social mobility during childhood [4], these findings highlight an opportunity for intervention during early life to produce more favorable immune response profiles. Carroll et al. recently reported that only very early SES (years 1–2 after birth) predicts circulating IL-6 in adults, raising the possibility that there may be a “critical window” in early childhood when SES conditions have a greater impact on future inflammation [54]. By investigating SES trajectories throughout childhood, our findings suggest that upward SES mobility during later childhood can potentially reverse the detrimental effects of low SES during this “critical window”. Notably in our study, the protective effect of increasing SES only applied to normal weight children.

Independent of SES trajectories, First Nations children were found to have 51% higher IL-6 responses compared to non-FN children. Since this association was also independent of overweight, and was found in FN children living both on and off reserve, we speculate that other genetic, lifestyle or environmental factors are responsible for the observed increase in IL-6 responses [59]. Consistent with this thesis, studies have reported that Canadian FN individuals have a higher frequency of single-nucleotide polymorphisms favouring increased expression of the IL-6 gene [43]. We add to this literature by presenting evidence for actual differences in immune cell production of IL-6 between Canadian FN and non-FN children.

This analysis also identifies another high risk group of children. Compared to their rural counterparts, children with atopic asthma in urban centers exhibited greater immune responsiveness. This finding is significant since there is an established connection between urban living and greater prevalence of inflammatory diseases such as asthma and ulcerative colitis [34], [35], but IL-6 differences according to residence location have not been widely studied. Recently, Huttenen et al. demonstrated increased toxicity and inflammatory responses in cultured macrophages exposed to airborne particles collected at urban versus rural sites [60]. Consistent with this study, our results suggest that innate immune activation (here, IL-6) could offer a biological marker of the “urban influence” on systemic inflammation, and the development of asthma or other chronic disease. More work will be required to test this hypothesis.

A major strength of this study was the determination in a large sample of children, of stimulated IL-6 responses to assess immune cell responsivity, rather than using a proxy measure of circulating IL-6 levels. Published serum or plasma concentrations typically range from <1 pg/ml to 2 pg/ml, a narrow range that is very close to the detection limit for most assays. Thus, many individuals have undetectable levels and reported differences can be challenging to interpret. In contrast, our *ex vivo* stimulation assay uses environmental levels of an innate immune ligand to generate reproducible IL-6 responses in every individual, with detectable levels over a broad range (10–10,000 pg/mL) yielding meaningful differences between subjects or groups. Our cohort included both asthmatic and healthy children from urban and rural environments, and comprised a sizeable group of FN children. Since child asthma status was accounted for in our analyses, the results can be generalized to children with and without asthma despite the case-control study design.

The major limitation of this study was that, in contrast to our ability to measure trajectories of SES, we were confined to a single determination of the IL-6 response at age 9. While one can speculate that genetic and early life environmental factors (such as family SES and residence location) preceded the development of immune phenotypes, direct evidence for this requires longitudinal immune studies over the first decade of life. Nevertheless, our single measure of IL-6 response is highly reliable since measurement error was controlled for by culturing PBMC in triplicate, stimulating with physiologic levels of LPS, performing IL-6 assays in duplicate, and utilizing standardized titration curves. Another potential limitation of our study is selection bias, since children without SES trajectory data were necessarily excluded. As shown in Table 1, this restriction led to under-representation of rural and overweight children and over-representation of children with maternal allergy/asthma; however, these factors were adjusted for in our multivariate analysis. More importantly, our main findings would be unaffected by this potential bias since children with and without SES trajectory data did not differ with respect to the main exposure (SES, indicated by current household income) or outcome (IL-6 production) of interest. While IL-6 responses, overweight and child atopy/asthma were measured directly, other variables were assessed by questionnaires, which are sensitive to bias [61]. Our questionnaires were expertly designed to minimize bias, and several of the measures have subsequently been validated against medical records [28]. Notably, SES trajectories were based on retrospective recall of housing data, which could create bias if data were recalled differently by parents of children with high or low immune responsiveness; however, this seems unlikely. Others have shown that housing data tend to be accurately recalled [55], and there is no reason to expect this accuracy to vary systematically according to child immune responsiveness. Finally, although we have tested a large number of potential covariates, we cannot exclude the possibility that other factors (beyond the scope of our analyses) may contribute to the pathway between SES trajectories, immune responsiveness and subsequent health outcomes.

In sum, using a novel strategy for investigating immune responsiveness in conjunction with novel SES trajectory measures, we discovered an association between chronic exposure to a low-SES environment from birth and elevated IL-6 responses at age 9, indicating excess innate immune activity and heightened risk for disease. Collectively, these results suggest a role for global innate immune activation in the well-established relationships between SES and clinical outcomes. Most importantly from the knowledge translation perspective, the data reveal a marked protective effect among normal-weight children whose families improved their SES during childhood. These findings provide experimental data in support of recent appeals [62] for interventions aimed at decreasing socioeconomic disparities during early childhood in order to limit or reverse the detrimental effects of exposure to low SES.
